# Electronic Health Literacy in Individuals with Chronic Pain and Its Association with Psychological Function

**DOI:** 10.3390/ijerph182312528

**Published:** 2021-11-28

**Authors:** Elena Castarlenas, Elisabet Sánchez-Rodríguez, Rubén Roy, Catarina Tomé-Pires, Ester Solé, Mark P. Jensen, Jordi Miró

**Affiliations:** 1 Universitat Rovira i Virgili, Department of Psychology, Research Center for Behavior Assessment (CRAMC), Chair in Pediatric Pain Universitat Rovira i Virgili-Fudación Grünenthal, Unit for the Study and Treatment of Pain—ALGOS, 43007 Tarragona, Catalonia, Spain; elena.castarlenas@urv.cat (E.C.); elisabet.sanchez@urv.cat (E.S.-R.); ruben.roy@urv.cat (R.R.); jamnista@gmail.com (C.T.-P.); ester.sole@urv.cat (E.S.); 2 Institut d’Investigació Sanitària Pere Virgili, 43007 Tarragona, Catalonia, Spain; 3 Department of Rehabilitation Medicine, University of Washington, Seattle, WA 98104, USA; mjensen@uw.edu

**Keywords:** eHealth literacy, chronic pain, self-efficacy, psychological function

## Abstract

Electronic health literacy skills and competences are important for empowering people to have an active role in making appropriate health care decisions. The aims of this cross-sectional study were to (1) examine the frequency of use of the Internet for seeking online information about chronic pain, (2) determine the level of eHealth literacy skills in the study sample, (3) identify the factors most closely associated with higher levels of eHealth literacy, and (4) examine self-efficacy as a potential mediator of the association between eHealth literacy and measures of pain and function in a sample of adults with chronic pain. One-hundred and sixty-one adults with chronic pain completed measures assessing internet use, eHealth literacy, pain interference, anxiety, depression, and pain-related self-efficacy. Results indicated that 70% of the participants are active users of the Internet for seeking information related to their health. The level of eHealth literacy skills was not statistically significantly associated with participants’ age or pain interference but was significantly negatively associated with both anxiety and depression. In addition, the findings showed that self-efficacy fully explained the relationship between eHealth literacy and depression and partially explained the relationship between eHealth literacy and anxiety. Self-efficacy should be considered as a treatment target in eHealth literacy interventions, due to its role in explaining the potential benefits of eHealth literacy.

## 1. Introduction

Chronic pain is a common health condition worldwide, associated with financial, physical, and emotional burdens [1,2,3,4]. It is also one of the most common reasons for individuals seeking health care [5]. Given the evidence that the severity and impact of chronic pain is associated with biological, psychological, and social variables, it is often managed with multidisciplinary and multicomponent programs that address all of these factors [6,7,8]. Among the effective components of most, if not all, pain treatment programs is pain education [9,10]. Pain education is designed to increase patients’ knowledge about pain, which is thought to lead to increases in adaptive coping responses and reductions in pain and its negative impact [11].

Specific knowledge domains addressed by pain education include information about the possible causes of pain, treatment options, effective self-management strategies, and prognosis [12]. Patients can obtain this information from a variety of sources, including the Internet [13]. For example, de Boer and colleagues [14] found that 39% of a sample of 200 patients attending a university pain center used the Internet to obtain information about their pain condition. However, although the Internet has become a common source of information about pain, the quality and usefulness of the information available on the Internet can be questioned [15,16]. 

Electronic health literacy (eHealth literacy) is a relatively new construct that extends the study of the traditional health literacy to encompass health literacy as it relates to the Internet. Specifically, eHealth literacy has been defined by Norman and Skinner as “…the ability to seek, find, understand, and appraise health information from electronic sources and apply the knowledge gained to addressing or solving a health problem” [17] (p. 2). Thus, eHealth literacy goes beyond the individual’s ability to simply obtain relevant information about health from the Internet. It also includes the ability to apply that information to one’s health care [18]. 

Although a considerable amount of research has examined traditional health literacy in individuals with chronic pain [19,20,21,22,23], and research on eHealth literacy has been conducted with individuals with other health conditions such as cancer, diabetics, epilepsy, cardiovascular diseases, recent fractures, or dental disease [24,25,26,27], none of those studies included pain-related variables as an outcome variables. As a whole, this research has found that being younger and having a higher level of education is associated with higher eHealth literacy skills [24,25,26,27,28]. Findings related to sex are less consistent; some studies have reported that eHealth literacy skills are similar between females and males [25], while others have found significant sex differences, with female predilection [24]. 

Research has also shown that having higher levels of eHealth literacy skills is associated with better health outcomes, as indicated by greater medication adherence, higher levels of quality of life and psychosocial well-being, and the adoption of adaptive health behaviors [29,30,31,32]. Self-efficacy, which is an individual’s judgment of his or her ability to engage in or perform a specific activity, is a key component in the conceptual framework study of eHealth literacy, and has been hypothesized to mediate the associations between eHealth literacy skills and health outcomes [33]. To our knowledge, however, only one study has examined self-efficacy as a potential mediator of the effects of eHealth literacy in chronic pain samples. Specifically, Rabenbauer and Mevenkamp [34] found that self-efficacy mediated the association between eHealth literacy skills and healthy habits (e.g., organized physical exercise) in a sample of 207 adults with chronic back pain who used eHealth interventions for the management of chronic pain. However, these initial findings have yet to be replicated.

Given these considerations, the current study had four primary aims. First, we sought to examine the frequency of use of the Internet for seeking online information about chronic pain. Second, we wanted to better understand the association between eHealth literacy skills related to pain (i.e., seeking and understanding information about pain, and applying this information to own pain problems) and measures of pain and function in a sample of adults with chronic pain. Third, we sought to identify the factors most closely associated with higher levels of eHealth literacy in the study sample. Finally, we sought to test pain-related self-efficacy as a possible mediator of the associations between eHealth literacy and measures of function. On the basis of the research published to date, we hypothesized that seeking health information online would be common in a substantial subset of participants (i.e., that 33% or more would report that they sought health information online about their problem [14]). We also hypothesized that the levels of eHealth literacy would be negatively associated with participants’ age and their levels of anxiety, depression, and pain interference. Finally, we hypothesized that self-efficacy would mediate the associations between eHealth literacy and anxiety, depression, and pain interference.

## 2. Materials and Methods

### 2.1. Participants

Study participants were recruited from the general population through sending an invitation via associations of patients or groups of patients with chronic pain in social networks. For individuals to be considered as potential participants, they had to (1) be aged 18 years old or older, (2) report having a pain problem of at least three months’ duration, (3) be able to understand Spanish, and (4) have access to an electronic device connected to the Internet to be able to respond to the online survey. 

Sample size estimation was calculated using G*Power [35]. The results revealed that at least 89 participants should be needed to address the study objectives with the planned analyses (effect size f^2^ = 0.15; α = 0.05 at 2-tailed; power = 0.95; two predictors).

### 2.2. Procedure

A cross-sectional study was conducted to address the objectives of the study. We created an online survey using the LimeSurvey program (https://www.limesurvey.org/es/, accessed on 24 November 2021) that included all the variables and instruments of interest for this study. The survey was made available during the months of November 2019 through January 2020. A short description of the study, which included a link for contacting research staff if a potential participant was interested in participating, was shared via social networks mainly through the profiles of associations of patients. We also encouraged individuals to share the study information through their own social networks to reach a wider audience. Once individuals clicked on the survey link, they could read additional details about the objectives and procedures of the study. The description of the study provided information about the study’s aims. Specifically, participants were told that the study aimed to examine what people do, feel, or think when they have pain. They were also informed that participation in this study was anonymous and voluntary. After providing their consent to participate, they were able to respond to the survey questions. Participants did not receive any compensation for completing the survey. As no follow-up was planned, we decided to collect responses anonymously. On average, participants spent 17 min to respond to the survey. Participants were requested to respond to each question in the survey. Responses from 18 individuals were excluded from the planned analyses due to their failing to respond to all questions (the completion rate in this study was 90%). 

All study procedures were approved by the Internal Review Board of the Universitat Rovira i Virgili. 

### 2.3. Measures

Demographic and descriptive variables: Participants were asked to provide information regarding their gender, age, and maximum education level. 

Pain information: We asked participants whether they had been experiencing pain for 3 or more months to ensure that their pain condition met the temporal criteria to be considered as chronic [36]. Participants were also asked to indicate the location of their most frequent pain problem, if they did or did not have a specific pain diagnosis, and if they were or were not on medical leave due to their pain problem(s).

Use of the Internet for seeking health information: Participants were asked to provide the frequency of their use of the Internet to seek information about their chronic pain condition, using a 5-point scale (1 = “never,” 2 = “almost never,” 3 = “sometimes,” 4 = “almost always,” and 5 = “always”). 

Health Literacy in an Electronic Context: We used a modified version of the 8-item eHealth Literacy Scale (eHEALS) [37] to assess the participants’ perception of their knowledge, comfort, and resources at finding, evaluating, and applying electronic health information related to their chronic pain problem(s). Specifically, we modified the original instructions slightly by asking participants to respond to the items on the scale with respect to their chronic pain health problem (sample items: “I know how to use the Internet to answer my health questions about chronic pain,” “I have the skills I need to evaluate the health resources about chronic pain that I find on the Internet”). Respondents indicated their level of agreement with each item using a 5-point Likert scale, ranging from 1 (“strongly disagree”) to 5 (“strongly agree”). Responses were summed to create a total score that can range from 8 to 40, with higher scores representing higher self-perceived eHealth literacy related to chronic pain. Previous studies have identified a score of 26 or higher on this scale as indicative of high eHealth literacy skills [38,39]. eHEALS scores have been shown to be reliable and valid in a wide range of populations and contexts [40,41,42], and the Spanish version was translated and shown to be valid by Paramio and colleagues [43]. The Cronbach’s alpha coefficient of the scale in the current sample was 0.94, indicating an excellent internal consistency.

Anxiety and depression symptom severity: The Hospital Anxiety and Depression Scale (HADS) originally developed by Zigmond and Snaith [44] was used to assess anxiety and depression symptoms severity. The questionnaire includes seven questions for assessing anxiety (HADS Anxiety) and seven for assessing depression (HADS Depression) symptom severity. Respondents were asked to indicate the frequency or severity (depending on the item) of each anxiety or depression symptom listed on a 4-point Likert scale (e.g., 0 = “not at all” or “only occasionally”; 3 = “very often” or “very much indeed”). The items of each subscale were summed to obtain a total score, which can range from 0 to 21; higher scores represent higher levels of depression or anxiety symptom severity. The Spanish version used in this study has been shown to provide valid and reliable scores [45]. In the current study, the internal consistency coefficients (Cronbach’s alpha) for the anxiety and the depression subscales were 0.88 and 0.87, respectively, indicating a good internal consistency for both.

Pain self-efficacy: We used the 10-item Pain Self-Efficacy Questionnaire (PSEQ) [46] to measure the confidence in performing activities despite pain. With the PSEQ, responders are asked to rate their level of confidence for performing at present each activity described on a 7-point Likert scale, where 0 = “not at all confident” and 6 = “completely confident”. The total PSEQ score is calculated by summing the responses; thus, the total score can range from 0 to 60, with higher scores indicating higher levels of pain self-efficacy. Scores on the PSEQ have shown good validity and reliability properties when used with samples of adults with chronic pain, including our population [47]. The Cronbach’s alpha in the current sample indicated excellent internal consistency (α = 0.94). 

Pain interference with daily activities: The Pain Interference Scale of the Brief Pain Inventory (BPI) [48] was used to assess the pain impact on functioning. The BPI has 7 items describing daily activities, and respondents are asked to indicate the extent that pain interfered with the activity in the past 24 h on a 0 (“does not interfere”) to 10 (“completely interferes”) scale. The total score is obtained by computing the mean for the seven items, resulting in an interference score that could range from 0 to 10. The BPI is a widely used tool with multiples studies supporting its reliability and validity [49,50,51,52], including studies with Spanish-speaking individuals [53,54,55]. The Cronbach’s alpha of the pain interference scale in the current sample was 0.92, indicating excellent internal consistency. 

### 2.4. Data Analysis

We first computed the means and standard deviations (continuous variables), as well as number and percentages (categorical variables) of the demographic and study variables to describe the sample and address the first two study aims (that is, to examine the frequency of use of the Internet for seeking online information about chronic pain and the level of eHealth literacy skills in the sample). We computed a Pearson correlation coefficient between participants’ age and scores on the eHEALS to test the hypotheses that these variables would be positively associated. We then computed Pearson correlation coefficients between the eHealth literacy score and the measures of pain interference, depression, and anxiety symptoms to test the third hypothesis, with the plan to only proceed with a formal mediational analysis if these associations were statistically significant (i.e., in order to ensure that there was an association to explain). Finally, we performed mediation analyses to test the hypothesized mediating role of pain-related self-efficacy between eHealth literacy (independent variable) and criterion variables using the PROCESS macro version 3.4 for SPSS developed by Hayes (available at https://www.processmacro.org, accessed on 24 November 2021). For bootstrap, 5000 samples were computed. All statistical analyses were performed using the Statistical Package for Social Sciences (SPSS) for Windows Version 23.

## 3. Results

### 3.1. Sample Description

Participants consisted of a sample of 161 adults with chronic pain problems who were recruited from the general population. The overwhelming majority of participants were women (*N* = 154, 96%), ranging in age from 24 to 68 years, with a mean age of 44.63 years (SD = 9.55). The most common pain sites were the lower back (*N* = 57, 35%), the shoulder, and the upper limbs (*N* = 48, 30%). Table 1 provides additional information about the participants. 

### 3.2. Internet Use for Seeking Health Information and eHealth Literacy Skills 

One-hundred and thirteen (70%) participants in our study reported that they used the Internet “almost always” or “always” for seeking information related to their chronic pain problem (specifically: never = 1 (1%), almost never = 3 (2%), sometimes = 44 (27%), almost always = 38 (24%), and always = 75 (46%) of the total participants). The mean score on the eHEALS scale for the whole sample was 29.53 (SD = 6.54; range = 9–40) and 42 (26%) of the participants had scores below 26, indicating low levels of eHealth literacy skills [38,39]. 

### 3.3. Association between eHealth Literacy Skills, Participants’ Age, and Health-Related Outcomes

No statistically significant association was found between participants’ age and the total score of the eHEALS scale (r = 0.02, *p* = 0.845). In addition, the measure of eHealth literacy was not statistically significantly associated with pain interference (r = −0.13, *p* = 0.113). However, it was significantly associated with both anxiety and depression (r’s = −0.23 and −0.24, respectively, *p*’s < 0.001). We therefore proceeded to evaluate the extent to which general self-efficacy mediated the association between eHealth literacy and anxiety and depression symptoms. 

### 3.4. Self-Efficacy as a Mediator of the Relationship between eHealth Literacy and Health-Related Outcomes 

**Figure 1:**
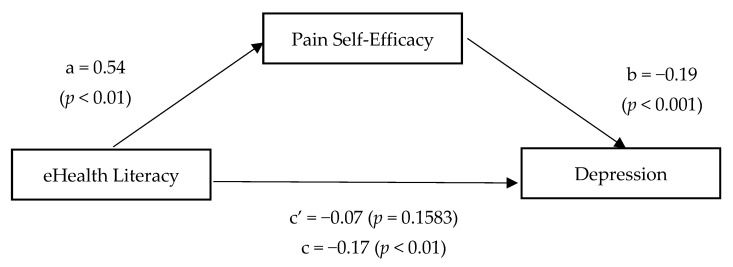
Relationship between eHealth literacy and depression mediated by pain-related self-efficacy.

Figure 1 depicts the results of the mediational analyses as hypothesized when depression is considered the criterion variable. Path a, that is, the effect of eHealth literacy (independent variable) on pain-related self-efficacy (mediator variable), was statistically significant (path a: β = 0.54, t = 3.09, *p* < 0.01). The data indicated a direct and negative association between pain-related self-efficacy and depressive symptoms (path b: β = −0.19, t = −9.16, *p* < 0.001). Moreover, the relationship between eHealth literacy and depression is fully explained by self-efficacy (path c: β = −0.17, t = −3.01, *p* < 0.01). Bootstrapping using confidence intervals not including zero confirmed that the mediating role of pain self-efficacy was statistically significant (β = 0.01, 95% confidence interval = −0.1722 to −0.0366, 5000 bootstrap resamples).

**Figure 2:**
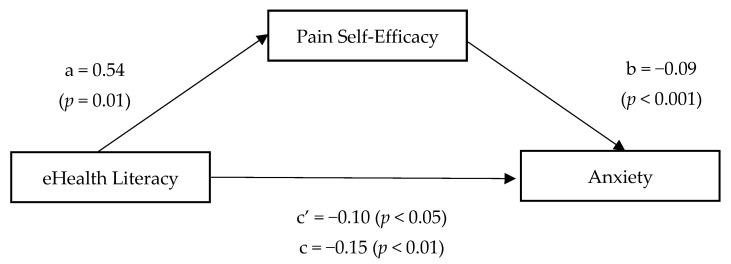
Relationship between eHealth literacy and anxiety mediated by pain-related self-efficacy.

A summary of the results of the mediational analyses when examining the role of pain-related self-efficacy as a mediator on the relation between eHealth literacy and anxiety symptoms is shown in Figure 2. The direct and significant effect of the independent variable on the mediator has been previously reported. We also found a negative and significant effect of pain-related self-efficacy on anxiety (path b: β = −0.09, t = −3.86, *p* < 0.001). Unlike the previous models, the effect of eHealth literacy on the criterion variable (i.e., anxiety) was found to be statistically significant (path c’: β = −0.10, t = −1.99, *p* < 0.05). In this case, the findings support partial mediation for pain-related self-efficacy on the relationship between the level of eHealth literacy and anxiety, that is, the indirect effect (path c: β = −0.15, t = −2.88, *p* < 0.01) explains part of the relationship between eHealth literacy and anxiety. Bootstrapping method with confidence intervals not including zero value confirms that the mediating role of pain self-efficacy was statistically significant (β = −0.05, 95% confidence interval = −0.0899 to −0.0126, 5000 bootstrap resamples).

## 4. Discussion

This study contributes knowledge about the eHealth literacy in individuals with chronic pain, as well as on the association between chronic pain-related eHealth literacy and function in these individuals. Consistent with the study hypothesis, a large number of participants in this sample (70%) were active in seeking information related to their chronic pain condition on the Internet. This percentage is considerably higher than that found by other authors [14] and it might be due to the characteristics of the sample. Participants in this study were recruited via social networks mainly through the profiles of associations of patients. Therefore, it seems likely that these participants were familiar with online resources, and that they were used to seek information on the Internet, health-related or otherwise. Moreover, more than 10 years has passed between de Boer and colleagues’ study and ours; in the last few years, internet use for obtaining information about health conditions has increased exponentially [56]. 

Most of the participants in this study (74%) obtained a score of 26 or higher on the eHEALS scale, indicating high levels of eHealth literacy skills on the basis of the cutoff used in previous studies [38,39]. In our study, the mean score of eHEALS was 29.5 (out of 40), which is similar to those reported by Richtening and colleagues [38] in a sample of 453 adults at risk for cardiovascular diseases, who found a mean score on the eHEALS of 27.2 and that 66% of their participants had a high level of eHealth literacy (≥26 out of 40). 

The results did not support the hypothesis that participants’ age would be negatively associated with higher eHealth literacy skills. Although the majority of previous studies have found that being younger is associated with a higher level of eHealth literacy [24,25,26,27,28], other researchers have obtained results similar to ours. For example, Milne and colleagues [57] did not find a statistically significant association between eHealth literacy and age in a sample of 83 primary lung cancer survivors. 

On the other hand, the hypothesis that eHealth literacy would be associated with health outcomes was partially supported. eHealth literacy was significantly and negatively associated with anxiety and depression but was not significantly associated with pain interference. Finally, the data partially supported the hypothesis that self-efficacy mediated the association between eHealth literacy and patient function (here, depression and anxiety, although not pain interference). As noted in the Introduction section, self-efficacy has been identified as a mediator of the association between eHealth literacy and health status both in clinical and community samples (e.g., [34,58]). Moreover, self-efficacy beliefs have been found to be a mediator in the association between measures of “traditional” health literacy skills and health-related outcomes (e.g., [59,60,61,62]). For example, Jones and colleagues [61] found that self-efficacy mediated the association between oral health literacy and self-rated oral health in a sample of 278 indigenous adults from South Australia. 

The results of this study have important research and clinical implications. First, this study contributes to a better understanding of the relationship between eHealth literacy and psychological health status in a sample of adults with chronic pain, a target population in which these associations have not yet been thoroughly explored. In addition, the direct associations found between measures of electronic literacy and psychological function highlight the potential importance of having adequate electronic literacy skills. This finding is consistent with the results of others that show that higher electronic literacy skills are more likely to be associated with better health-related outcomes [63]. 

With respect to the clinical implications of the study findings, eHealth literacy encompasses a set of abilities that can be learned. The current studies suggest that eHealth literacy interventions should emphasize increases in self-efficacy as a component of intervention, in order to maximize the potential benefits of the intervention on psychological function. Research to evaluate the efficacy of treatments that could enhance eHealth literacy, as well as to enhance self-efficacy for using the Internet in adaptive ways to better manage pain, is warranted. Although some eHealth literacy interventions have demonstrated benefits for patients and community samples, more efforts are required to develop these interventions in ways that are informed by eHealth literacy conceptual models [33]. This is of special relevance when considering that high levels of eHealth literacy skills have been found to be associated with variables that predict better treatment outcomes, such as treatment adherence, motivation, adaptive health behaviors, and the degree of trust in health care providers [64,65]. It is also possible that treatments which target non-eHealth literacy skills as primary outcomes—such as those that target depression or anxiety—might have an indirect impact on eHealth skills, which could then help to maintain treatment gains. Research examining this possibility is also warranted.

A number of limitations of this study should be considered when interpreting the results. First, the sample was composed by adults with chronic pain problems recruited from patients’ associations with an active role in social networks who were responding to an online survey. Thus, the extent to which they generalize to other adults with chronic pain who would not be interested or willing to participate in a study such as this one, or to adults with chronic pain seen in clinics and health care centers (i.e., patients), is not known. Future studies should be conducted with other samples of individuals with chronic pain to help determine the generalizability of the findings. Second, and also related to the characteristics of the sample, almost all the participants in our study were females. As a result, we were not able to examine gender-related differences in the variables studied. Research is needed to study eHealth literacy in more balanced samples and looking at other variables that could help explain the quality of the experience in using the Internet for health-related purposes among individuals with chronic pain. For example, it would have been interesting to include some additional measures examining the attitudes and experience in the use of electronic sources for seeking health-related information, beyond the frequency in which they do that. Further research needs also to examine the role of the eHealth literacy skills as moderators between sociodemographic variables (e.g., socioeconomic or education level) and health care habits and outcomes. 

## 5. Conclusions

This study provides new information about eHealth literacy and its association with psychosocial variables in individuals with chronic pain. The data showed that seeking information about health is a common practice. However, contrary to what was hypothesized, participants’ age was not significantly associated with eHealth literacy. In addition, the findings of this study showed the potential role of this literacy on emotional symptoms and the role of self-efficacy as a mediator between eHealth literacy and function in adults with chronic pain. Future efforts should be focused on the development and assessment of effective educational programs which enhance electronic health literacy in individuals with chronic health conditions as well as for the population from the community. Further studies examining the association between eHealth literacy and function and its mediators are also warranted.

## Figures and Tables

**Table 1:** Descriptive characteristics of the study participants.

Descriptive Characteristic		
Participants (*N*)		161
Mean age in years (range; SD)		44.63 (24–68; 9.55)
Gender, *N* (%)	Female	154 (96)
	Male	7 (4)
Education level, *N* (%)	Did not complete primary education	63 (39)
	Completed primary education	20 (12)
	Completed secondary education	52 (32)
	Completed bachelor’s degree	17 (11)
	Post-bachelor education	4 (3)
On medical leave due to pain? *N* (%)	No	113 (70)
	Yes	48 (30)
Have a specific pain diagnoses? *N* (%)	No	17 (11)
	Yes	144 (89)
Location of the most frequent chronic pain, *N* (%)	Head, face, and mouth	11 (7)
	Cervical region	17 (11)
	Upper shoulder and upper limbs	48 (30)
	Thoracic region	4 (3)
	Abdominal region	3 (2)
	Lower back, lumbar spine, sacrum, and coccyx	57 (35)
	Lower limbs	2 (1)
	Pelvic region	2 (1)
	Anal, perineal, and genital region	17 (11)

## Data Availability

The dataset used and analyzed in this study is available from the corresponding author upon request.
